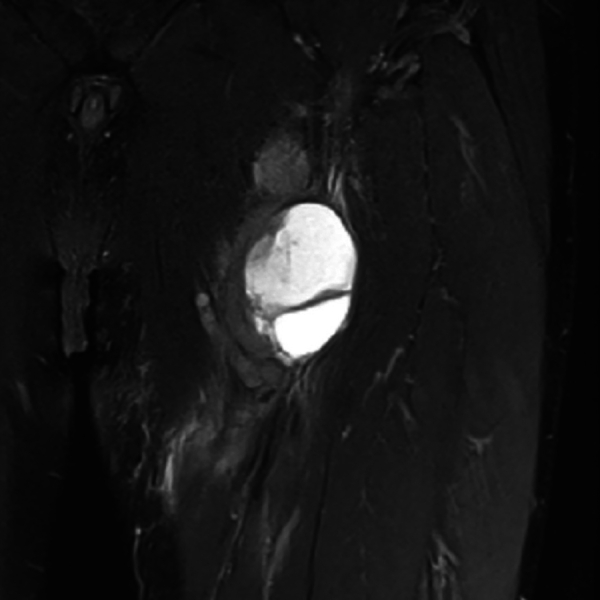# An initially misdiagnosed case of leiomyosarcoma originated from the femoral vein

**DOI:** 10.1016/j.jvsv.2026.102523

**Published:** 2026-05-22

**Authors:** Xiaoliang Yin, Dehai Lang, Songjie Hu

**Affiliations:** Department of Vascular Surgery, Ningbo No.2 Hospital, Wenzhou Medical University, Ningbo, Zhejiang Province

A 56-year-old man presented with vascular leiomyosarcoma (LSM), accompanied by pain and discomfort in the left groin. Ultrasound examination of the leg showed a mixed echogenic mass located outside the left femoral vein. Contrast-enhanced magnetic resonance imaging revealed a mixed-signal mass adjacent to the left femoral vein, accompanied by iliac and femoral vein thrombosis, with consideration of tumor-related lesions (Cover/*A*). The patient had initially received anticoagulant therapy and percutaneous mechanical thrombectomy 1 year earlier because of sudden swelling of left lower limb, ultrasound evidence of thrombosis in the left femoral vein, and an elevated D-dimer level. During surgery, the femoral artery, vein, and nerve were found to be encased by the tumor. Fish-flesh-like tissue was observed within and around the femoral vein, but no thrombus was detected inside the femoral vein (*B*). The tissue was sent for pathological examination.

Radical resection was carried out after frozen section pathology indicated a spindle cell tumor that was at least a low-grade malignant. The femoral artery, vein, and nerve were removed along with the tumor and the surrounding muscle tissue (*C*). The ipsilateral great saphenous vein was used for in situ revascularization of the femoral artery (*D*). Postoperative pathology confirmed high-grade sarcoma, and immunohistochemistry validated the diagnosis of smooth muscle sarcoma originating from the vasculature.

Postoperatively, the patient underwent radiotherapy and chemotherapy. After 20 months, a computed tomography scan showed several soft tissue masses surrounding the left iliac artery, several enlarged lymph nodes in the inguinal region, and possible recurrence of the tumor. Pleural effusion and several metastatic lesions were identified in the lungs, after which the patient discontinued further treatment.

LSM accounts for approximately 2% of all sarcomas.^1^ Compared with other sarcoma types, LSM generally has a poorer prognosis, and early diagnosis is often challenging. Clinical manifestations vary depending on the affected vessel, tumor growth rate, and the presence of thrombosis. These may range from localized symptoms to distant metastases.^2^ In this case, the LSM originated from the femoral vein, but was initially misdiagnosed as deep venous thrombosis. Pathological examination later confirmed the diagnosis of smooth muscle sarcoma arising from the femoral vein. The purpose of this case report was to raise awareness of this rare disease. Written informed consent was obtained from the patient for reporting of the case details and imaging studies.

**Figure:**
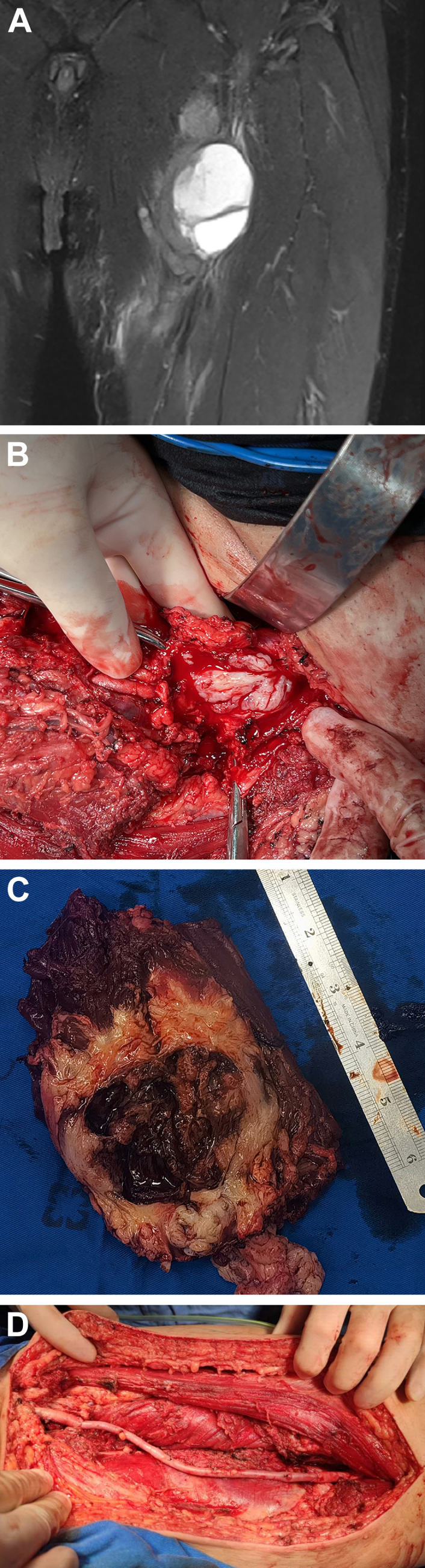

## Availability of data and materials

All data generated or analysed during this study are included in this published article.

## Funding

Supported by 10.13039/501100014759Medical Scientific Research Foundation of Zhejiang Province, China (Project No. 2024KY1559, No. 2024KY1558, and No. 2024KY1549), Clinical Key Specialty Construction Project of Zhejiang Province (No. 2026004), NINGBO Leading Medical & Health Discipline (No. 2026-A25).

## Disclosures

None.

## Figures and Tables

**Cover image:**